# Virtual screen for repurposing approved and experimental drugs for candidate inhibitors of EBOLA virus infection

**DOI:** 10.12688/f1000research.6110.1

**Published:** 2015-02-02

**Authors:** Veljko Veljkovic, Philippe M. Loiseau, Bruno Figadere, Sanja Glisic, Nevena Veljkovic, Vladimir R. Perovic, David P. Cavanaugh, Donald R. Branch

**Affiliations:** 1 Center for Multidisciplinary Research, University of Belgrade, Institute of Nuclear Sciences VINCA, P.O. Box 522, 11001 Belgrade, Serbia; 2 Antiparasitic Chemotherapy, UMR 8076 CNRS BioCIS, Faculty of Pharmacy Université Paris-Sud, Rue Jean-Baptiste Clément, F 92290- Chatenay-Malabry, France; 3 Bench Electronics, Bradford Dr., Huntsville, AL, 35801, USA; 4 Canadian Blood Services, Center for Innovation, 67 College Street, Toronto, Ontario, M5G 2M1, Canada

**Keywords:** Ebola virus, drug candidates, entry inhibitors, virtual screening

## Abstract

The ongoing Ebola virus epidemic has presented numerous challenges with respect to control and treatment because there are no approved drugs or vaccines for the Ebola virus disease (EVD). Herein is proposed simple theoretical criterion for fast virtual screening of molecular libraries for candidate inhibitors of Ebola virus infection. We performed a repurposing screen of 6438 drugs from DrugBank using this criterion and selected 267 approved and 382 experimental drugs as candidates for treatment of EVD including 15 anti-malarial drugs and 32 antibiotics. An open source Web server allowing screening of molecular libraries for candidate drugs for treatment of EVD was also established.

## Introduction

The current Ebola virus outbreak is one of the largest outbreaks of its kind in history and the first in West Africa. By January 14, 2015, a total of 21296 probable and confirmed cases, including 8429 deaths from Ebola virus disease (EVD), had been reported from five countries in West Africa - Guinea, Liberia, Nigeria, Senegal, and Sierra Leone ( http://apps.who.int/iris/bitstream/10665/148237/2/roadmapsitrep_14Jan2015_eng.pdf?ua=1). EVD with a high case-fatality rate of 40% and with currently no approved vaccine or therapy, represents a major public health threat.

In response to the current Ebola virus outbreak, the international community has urged for accelerated development of drugs against EVD but also has endorsed the clinical use of unregistered treatments for Ebola ^1^. Conventional time and the money consuming approach of drug development (> 10 years; > 2 billions $) does not meet the current urgent need for anti-Ebola drugs. Repurposing or repositioning of existing drugs could overcome some of these obstacles and help in the rapid discovery and development of therapeutics for EVD, although this approach does not negate the need for some preclinical studies and clinical trials for validation of the proposed indications. Recently, results of two large repurposing screenings of Food and Drug Administration (FDA)-approved drugs have been reported. In the first study, Madrid and co-workers performed *in vitro* and *in vivo* (in mice) screening of 1012 FDA-approved drugs and selected 24 candidate entry inhibitors for Ebola virus ^2^. In the second study, 53 inhibitors of Ebola virus infection with IC _50_ < 10 µM and selectivity index SI > 10-fold have been identified by *in vitro* screening of 2816 FDA-approved drugs ^3^. In the same study, an additional 95 drugs which are active against Ebola virus infection with IC _50_ > 10 µM and SI <10-fold were also reported.

Although *in vitro* and *in vivo* screening for repurposing/repositioning of existing drugs could significantly accelerate discovery of new drugs these approaches are time-consuming and costly for screening of large drug libraries. Recently, we proposed a novel approach for *in silico* screening of molecular libraries for drug candidates ^4– 8^. This approach, which uses the average quasi valence number (AQVN) and the electron-ion interaction potential (EIIP), parameters determining long-range interaction between biological molecules, might hold a key to overcoming some of these obstacles in experimental screening by significantly reducing the number of compounds which should be *in vitro* and *in vivo* tested ^9^.

Herein, 267 approved and 382 experimental drugs, selected by the EIIP/AQVN-based virtual screening of DrugBank ( http://www.drugbank.ca), have been proposed as candidate drugs for treatment of EVD. An open access portal allowing screening of molecular libraries for candidate drugs for treatment of EVD was established.

## Material and methods

### Molecular libraries

For screening of drugs for repurposing to select candidates for Ebola virus entry inhibitors, 1463 approved and 4975 experimental drugs from DrugBank ( http://www.drugbank.ca) were screened. For development of the predictive criterion used in this analysis, the learning set ( Dataset 1) encompassing 152 drugs which are selected as inhibitors of Ebola virus infection by *in vitro* and *in vivo* screening of 3828 FDA-approved drugs ^2, 3^, was established. As control data sets 45,010,644 compounds from PubChem ( http://www.ncbi.nlm.nih.gov/pccompound) and 49 Ebola virus entry inhibitors collected by data mining of literature and patents, were used. For screening of literature data the NCBI literature database PubMed ( http://www.ncbi.nlm.nih.gov/pubmed) was used. For search of patents and patent applications we used the Free Patent Online browser ( http://www.freepatentsonline.com).

### Drug repurposing screen to identify active compounds that block Ebola entry

Specific recognition and targeting between interacting biological molecules at distances > 5Å are determined by the average AQVN and the EIIP ^10^, which are derived from the general model pseudopotential ^11, 12^. These parameters for organic molecules are determined by the following simple equations ^10^:

$$EIIP=0.25\frac{Z^{*}\sin(1.04\;\pi \;Z^{*})}{2\pi }\;\;\;\;\;\;\;(1)$$

Where Z* is the average quasi-valence number (AQVN) determined by

$$Z^{*}=\frac{1}{N}\sum_{i=1}^{m}n_{i}Z_{i}\;\;\;\;\;\;\;\;\;\;\;\;\;\;\;\;\;(2)$$

where Z _*i*_ is the valence number of the *i*-th atomic component, n _*i*_ is the number of atoms of the *i*-th component, *m* is the number of atomic components in the molecule, and N is the total number of atoms. EIIP values calculated according to equation 1 and equation 2 are expressed in Rydberg units (Ry).

Among 3300 currently used molecular descriptors, AQVN and EIIP represent the unique physical properties which characterize the long-range interactions between biological molecules ^10^. Small molecules with similar AQVN and EIIP values interact with the common therapeutic target, which allow establish criterions for virtual screening of molecular libraries for compounds with similar therapeutic properties ^4– 9^. Here we develop the EIIP/AQVN-based criterion for virtual screening of molecular libraries for candidate drugs against Ebola virus infection.

## Results and discussion

**Figure 1.**
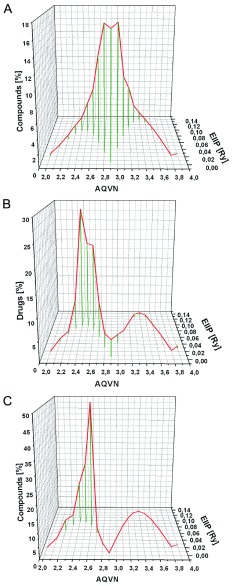
Distribution of compounds according to their average quasivalence number (AQVN) and electron-ion interaction potential (EIIP) values. ( **A**) 45010644 compounds from the PubChem database ( http://www.ncbi.nlm.nih.gov/pccompound); ( **B**) FDA-approved drugs which are active against Ebola virus infection ( Dataset 1) ^2, 3^; ( **C**) Entry inhibitors of Ebola virus ( Table 1).

Previously, analyses of the EIIP/AQVN distribution of 45,010,644 compounds from the PubChem database ( http://www.ncbi.nlm.nih.gov/pccompound) revealed that 92.5% of presented compounds are homogenously distributed within EIIP and AQVN intervals (0.00 – 0.11 Ry) and (2.4 – 3.3), respectively). This domain of the EIIP/AQVN space, encompassing the majority of known chemical compounds, is referred to as the “basic EIIP/AQVN chemical space” (BCS) ^6^. Analysis of the molecular training set ( Dataset 1), encompassing 152 small molecule inhibitors of Ebola virus infection selected by *in vitro* screening of 3828 FDA approved drugs ^2, 3^, show that 79% of these compounds are placed within AQVN and EIIP region (2.3 – 2.7) and (0.0829 – 0.0954 Ry), respectively (“Ebola Virus Infection Inhibitors Space”, EVIIS). The AQVN region (2.36 – 2.54) and the EIIP region (0.0912 – 0.0924 Ry) form the part of EVIIS which encompasses 55.5% of all drugs from the learning set (core EVIIS, cEVIS). Literature data mining reveals 49 compounds with experimentally proved activity against Ebola virus infection ( Table 1) ^13– 29^. Most of these compounds 47 (95.9%) are placed within EVIIS ( Table 1). Of note is that EVIIS and cEVIIS domains contain only 14.6% and 6.5% of compounds from PubChem, respectively. This confirms high specificity of clustering of Ebola virus infection inhibitors within the EIIP/AQVN space. Comparison of distributions of Ebola virus infection inhibitors and compounds from PubMed is given in Figure 1.

**Table 1.** Small-molecule entry inhibitors for Ebola virus.

Compound	Formula	AQVN	EIIP [Ry]	Reference
Chloroquine	C18H26ClN3	2.375	0.0941	16
Bafilomycin A1	C35H58O9	2.471	0.0960	17
Cytochalasin B	C29H37NO5	2.611	0.0810	17
Cytochalasin D	C30H37NO6	2.676	0.0672	17
Latruculion A	C22H31NO5S	2.667	0.0693	17
Jasplakinolide	C36H45BrN4O6	2.674	0.0676	18
Clomiphene	C26H28ClNO	2.526	0.0926	18
Toremifene	C26H28ClNO	2.526	0.0926	18
Chlorpromazine	C17H19ClN2S	2.600	0.0829	19
Amiodarone	C25H29I2NO3	2.567	0.0880	20
Dronedarone	C31H44N2O5S	2.578	0.0864	20
Verapamil	C27H38N2O4	2.535	0.0917	20
Clomiphene	C26H28ClNO	2.526	0.0926	21
AY-9944	C22H28Cl2N2	2.370	0.0938	21
Ro 48-8071	C23H27BrFNO2	2.505	0.0940	21
U18666A	C25H41NO2	2.290	0.0849	21
Terconazole	C26H31Cl2N5O3	2.687	0.0644	21
Triparanol	C27H32ClNO2	2.508	0.0941	21
Impramine	C19H24N2	2.444	0.0964	22
3.47	C34H43N3O5	2.635	0.0763	23
Cytochalasin B	C29H37NO5	2.611	0.0810	24
Cytochalasin D	C30H37NO6	2.676	0.0672	24
Latrunculin A	C22H31NO5S	2.667	0.0693	24
Jasplakinolide	C36H45BrN4O6	2.674	0.0676	24
NSC62914	C31H40O3	2.460	0.0962	25
Compound 1	C30H38N6O2	2.632	0.0770	26
Compound 2	C32H46N6	2.429	0.0963	26
Compound 3	C28H34N6O2	2.686	0.0635	26
Compound 5	C42H58N10O6	2.690	0.0635	27
Compound 8a	C17H23N3O3	2.695	0.0621	27
Compound 8b	C17H23N3O3	2.695	0.0621	27
Compound 8y	C16H20BrNO2	2.610	0.0812	27
Compound 15h	C15H20JN5O	2.667	0.0693	27
Compound 15k	C15H128Br3N5O	2.667	0.0693	27
Retinazone	C38H56Na3N5S2	2.385	0.0947	28
Compound 7	C17H12F4N2	2.467	0.0647	29
Brincidofovir *	C27H52N3O7P	2.467	0.0961	30
Hit compound 3	C25H35N3O2	2.494	0.0950	31
Hit compound 3.1	C21H24ClN3O2	2.667	0.0693	31
Hit compound 3.2	C20H29N3O2	2.518	0.0933	31
Hit compound 3.3	C30H35N3O2	2.556	0.0894	31
Hit compound 3.4	C25H32N4O3	2.656	0.0717	31
Hit compound 3.5	C20H23N3O2	2.708	0.0587	31
Hit compound 3.6	C25H33N3O2	2.540	0.09913	31
Hit compound 3.7	C22H27N3O3	2.691	0.0633	31
Hit compound 3.18	C26H37N3O2	2.471	0.0471	31
Hit compound 3.48	C34H43N3O5	2.635	0.0763	31
Hit compound 3.105	C34H40N6O2	2.658	0.0712	31
NSC 62914	C31H39O3	2.480	0.0957	32

*Experimental drug applied for treatment of Ebola patients in Liberia ( http://www.ox.ac.uk/news/2014-11-13-oxford-lead-trial-experimental-drug-ebola-patients)

FDA-approved drugs which are active against Ebola virus infection ^2, 3^ AQVN: average quasivalence number; EIIP: electron-ion interaction potential

It was shown that Ebola virus glycoprotein (GP)-mediated entry and infection is subordinated with a membrane-trafficking event that translocates a GP binding partner to the cell surface, which depends on microtubules ^30, 31^. Consistently, microtubule inhibitors which block this trafficking process could decrease infection without interfering with the direct binding and translocation of the Ebola virus into cells. AQVN and EIIP values of microtubule modulators and transcription inhibitors with reported anti-Ebola virus activity are given in Table 2. As can be seen, all these compounds, which do not directly affect binding and internalization of Ebola virus, are located outside of EVIIS. This additionally confirms the specificity of the EVIS domain.

**Table 2.** Viral transcription inhibitors and microtubule modulators with anti-Ebola virus activity.

Compound	Formula	AQVN	EIIP [Ry]
**Viral transcription inhibitors**			
BCX4430	C11H15N5O3	3.000	0.0439
Favipiravir	C5H4FN3O2	3.467	0.1304
C-c3Ado	C12H16N4O3	2.914	0.0112
c3Nep	C12H14N4O3	3.030	0.0552
“D-like” 1’-6’-isoneplanocin	C11H12N5O3	3.194	0.1076
“L-like” 1’-6’-isoneplanocin	C11H12N5O3	3.194	0.1076
CMLDBU3402	C30H26BrN3O7	3.045	0.1343
**Microtubule modulators**			
Vinblastine	C13H8Cl2N2O4	3.310	0.0130
Vinorelbine	C45H54N4O8	2.721	0.0552
Vincristine	C46H56N4O10	2.759	0.0439
Colchicine	C22H25NO6	2.852	0.0121
Nocodazole	C14H11N3O3S	3.312	0.1298
Mebendazole	C16H13N3O3	3.143	0.0934
Albendazole	C12H15N3O2S	2.909	0.0092

In further analysis we used EVIIS as a filter for virtual screening for candidate Ebola virus infection inhibitors. In Dataset 2 622 approved and 1089 experimental drugs in Dataset 3 selected by EVIIS screening of 6532 drugs from DrugBank are reported. Using cEVIIS, we located 267 approved and 382 experimental drugs. This small molecular library represents a source of candidate drugs for treatment of Ebola virus disease (EVD), which can be further experimentally tested.

Approved and experimental drugs selected as candidate for treatment of EVD AQVN: average quasivalence number; EIIP: electron-ion interaction potential

Experimental drugs selected as candidate for treatment of EVD AQVN: average quasivalence number; EIIP: electron-ion interaction potential

Madrid and co-workers selected 24 drugs by *in vitro* screening of 1012 FDA-approved drugs, which are effective against Ebola virus infection ^2^. They also showed that among these compounds, four antimalarial drugs (chloroquine, hydroxychloroquine, amodiaquine and aminoquinoline-13) also are effective against Ebola virus infection *in vivo* ^2^. Among 53 compounds which effectively inhibit Ebola virus infection *in vitro*, which Kouznetsova and co-workers selected from 2816 approved drugs, are also three anti-malarial drugs (mefloquione, chloroquine, amodiaquine) ^3^. It was also suggested that application of chloroquine for prevention of virus transmission should be considered because this compound significantly inhibits Ebola virus infection ^13^. Our analysis showed that 15 of 22 approved ant-malarial drugs ( http://en.wikipedia.org/wiki/Antimalarial_medication) are located in EVIIS ( Table 3). Six 2-alkylquinolines have been also included in this study. This chemical series is promising as some derivatives exhibited antiviral activity such as 2PQ, and 2QQ ^32, 33^ antimalarial activity such as 2PQ and 2PentQ2 ^34^, antileishmanial activity such as 2PQ ^35, 36^ and neurotrophin-like activity on dopaminergic neurons such as 2QI15 ^37^. These compounds exhibit some advantages in regard to their chemical synthesis with few steps and good yields as well as their chemical stability in tropical conditions of storage. Their combined effects against virus and Leishmania parasites suggested they could be an advantage for the treatment of Leishmania/HIV co-infections and they were considered as attractive enough to enter the pipeline of DNDi on 2010.

All these data strongly suggest that this class of drugs should be further investigated as a promising source of therapeutics for treatment of EVD. Anti-malarial drugs with dual activity should be of special interest because malaria represents the highest health-related disease in African countries with EVD.

Among 3828 FDA-approved drugs screened for anti-Ebola activity were six antibiotics which inhibit Ebola virus infection (azthromycin, erythromycin, spiramycin, dirithromycin, maduramicin, charitromycin) ^2, 3^. All these antibiotics are within EVIIS and four of them are in cEVIIS. Analysis of 184 approved antibiotics ( Dataset 4) showed that only 32 (17.4%) have AQVN and EIIP values in EVIIS, and that 11 of them are located within cEVIIS. Previously we reported domains of AQVN and EIIP which characterize different classes of antibiotics ( Table 4) ^6^. According to these data, among antibiotics some macrolides, pleuromutilins and aminoglycosides have the highest chance for inhibition of Ebola virus infection. Of note is that five of six antibiotics with experimentally proved activity against Ebola virus infection (azthromycin, erythromycin, spiramycin, dirithromycin, charitromycin) are macrolides. Antibiotics representing candidate Ebola virus infection inhibitors selected by EIIP/AQVN criterion are given in Table 5.

**Table 3.** Approved anti-malarial drugs selected as candidate drugs for EVD.

Compound	Formula	AQVN	EIIP [Ry]
Quinine	C20H24N2O2	2.625	0.0784
Chloroquinine	C18H26ClN3	2.375	0.0941
Amodiquinine	C20H22ClN3O	2.638	0.0756
Proguanil	C11H16ClN5	2.606	0.0819
Mefloquine	C17H16F6N2O	2.524	0.0928
Primaquine	C15H21NO3	2.600	0.0829
Halofantrine	C26H30Cl2F3NO	2.381	0.0945
Clindamycin	C18H33ClN2O5S	2.533	0.0919
Artemether	C16H26O5	2.553	0.0897
Piperaquine	C29H32Cl2N6	2.609	0.0814
Artemotil	C17H28O5	2.520	0.0931
Dihydroartemisin	C15H24O5	2.591	0.0844
Quinidine	C20H24N2O2	2.625	0.0784
Cinchonidine	C19H22N2O	2.591	0.0844
Artemisin	C15H22O5	2.667	0.0693

**Table 4.** AQVN and EIIP range of different antibiotics classes ^6^.

Antibiotic class	AQVN	EIIP [Ry]
Penicillins	2.975 - 3.180	0.035 - 0.124
Cephalosporins	3.071 - 3.473	0.070 - 0.130
Carbapenems & Penems	2.973 - 3.059	0.022 - 0.066
Monobactams	3.166 - 3.581	0.100 - 0.134
Quinolines	2.760 - 3.060	0.003 - 0.065
Aminoglycosides	2.552 - 2.820	0.024 - 0.084
Tetracyclines	2.933 - 3.111	0.018 - 0.084
Macrolides	2.467 - 2.630	0.077 - 0.096
Pleuromutilins	2.395 - 2.473	0.095 - 0.096
Nitrofurans	3.652 - 3.826	0.010 - 0.086

Approved antibiotics screened for candidate anti-Ebola drugs AQVN: average quasivalence number; EIIP: electron-ion interaction potential

Previous, we determined AQVN and EIIP domains characterizing different classes of anti-HIV drugs ^4– 9^. As can be seen in Table 6, the EIIP/AQVN domain of CCR5 HIV entry inhibitors is within EVIIS, and domains of CXCR4 HIV entry inhibitors and HIV protease inhibitors partially overlaps EVIIS. The EIIP/AQVN domains of other classes of anti-HIV agents are located outside EVIIS. This indicates that some HIV entry inhibitors and HIV protease inhibitors could also be effective drugs against Ebola virus infection.

**Table 5.** Antibiotics selected as candidate drugs for EVD.

Antibiotics	Formula	AQVN	EIIP [Ry]
Tiamulin	C _28_H _47_NO _4_S	2.395	0.095
Retapamulin	C _30_H _47_NO _4_S	2.434	0.096
Valnemulin	C _31_H _52_N _2_O _5_S	2.440	0.096
Azithromycin	C _38_H _72_N _2_O _12_	2.468	0.096
BC-3205	C _32_H _51_N _2_O _5_S	2.472	0.096
Dirithromycin	C _42_H _78_N _2_O _14_	2.500	0.095
Clarithromycin	C _38_H _69_NO _13_	2.512	0.094
Surfactin	C _53_H _93_N _7_O _13_	2.518	0.093
Erythromycin	C _37_H _67_NO _13_	2.525	0.093
Clindamycin	C _18_H _33_ClN _2_O _5_S	2.533	0.092
Roxithromycin	C _41_H _76_N _2_O _15_	2.537	0.092
Oleandomycin	C _35_H _61_NO _12_	2.550	0.090
Gentamicin	C _21_H _43_N _5_O _7_	2.553	0.090
Spiramycin	C _43_H _74_N _2_O _14_	2.556	0.089
Mupirocin	C _26_H _44_O _9_	2.557	0.089
Lincomycin	C _18_H _34_N _2_O _6_S	2.590	0.085
Netilmicin	C _21_H _41_N _5_O _7_	2.595	0.084
Astromicin	C _17_H _35_N _5_O _6_	2.603	0.082
Tylosin	C _46_H _77_NO _17_	2.610	0.081
Kitasamycin	C _35_H _59_NO _13_	2.611	0.081
Josamycin	C _42_H _69_NO _15_	2.614	0.080
Telithromycin	C _43_H _65_N _5_O _10_	2.618	0.080
Telithromycin	C _43_H _65_N _5_O _10_	2.618	0.080
Verdamicin	C _20_H _39_N _5_O _7_	2.620	0.080
Midecamycin	C _41_H _67_NO _15_	2.629	0.078
Troleandomycin	C _41_H _67_NO _15_	2.629	0.078
Sisomicin	C _19_H _37_N _5_O _7_	2.647	0.074
Cethromycin	C _42_H _59_N _3_O _10_	2.649	0.073
Carbomycin A	C _42_H _67_NO _16_	2.667	0.069
Dibekacin	C _18_H _37_N _5_O _8_	2.676	0.067
Echinocandin B	C _52_H _81_N _7_O _16_	2.692	0.063
Rifabutin	C _46_H _62_N _4_O _11_	2.699	0.061

**Table 6.** AQVN and EIIP range of anti-HIV drugs ^6^.

Target	AQVN	EIIP [Ry]
CXCR4	2.16 - 2.53	0.062 - 0.096
CCR5	2.42 - 2.63	0.079 - 0.099
PI	2.61 - 2.78	0.040 - 0.080
NRTI/NtRTI	2.92 - 3.20	0.040 - 0.100
INI	3.00 - 3.20	0.044 - 0.116
Anti-HIV flavonoids	3.34 - 3.59	0.110 - 0.135

In conclusion, the presented results show that the EIIP/AQVN criterion can be used as an efficient filter in virtual screening of molecular libraries for candidate inhibitors of Ebola virus infection. Approved ( Dataset 2) and experimental drugs ( Dataset 3), anti-malarial drugs ( Table 3) and antibiotics ( Table 5) selected by this criterion represents a valuable source of candidate therapeutics for treatment of EVD, some of which are already approved by FDA for treatment of other diseases which can be repurposed for use in EVD. We hope that these data, obtained by an *in silico* drug repurposing screen, will accelerate discovery of drugs for treatment of EVD, which are necessary in this ongoing emergency situation caused by the current unprecedented Ebola virus outbreak. To enable other researchers working on online EIIP/AQVN-based screening of different sources of small molecules for candidate Ebola drugs, we established an open web server ( http://www.biomedconsulting.info/ebola_screen.php).

## Data availability

The virtual screen for candidate inhibitors of EBOLA virus infection web tool is available at: http://www.biomedconsulting.info/tools/ebolascreen.php. An archived version can be accessed at: http://www.webcitation.org/6Vxtuojgx ^38^

F1000Research: Dataset 1. FDA-approved drugs which are active against Ebola virus infection ^2, 3^, 10.5256/f1000research.6110.d42876 ^39^

F1000Research: Dataset 2. Approved and experimental drugs selected as candidate for treatment of EVD, 10.5256/f1000research.6110.d42877 ^40^

F1000Research: Dataset 3. Experimental drugs selected as candidate for treatment of EVD, 10.5256/f1000research.6110.d42878 ^41^

F1000Research: Dataset 4. Approved antibiotics screened for candidate anti-Ebola drugs, 10.5256/f1000research.6110.d42879 ^42^
