# Supplementary material for: Virtual screen for repurposing approved and experimental drugs for candidate inhibitors of EBOLA virus infection
Source: F1000Res. 2015 Feb 2;4:34. [Version 1] doi: 10.12688/f1000research.6110.1 (PMC4329668; doi:10.12688/f1000research.6110.1)
Supplement: Peer review history

# Peer review: Referee response for version 1

**DOI:** 10.5256/f1000research.6544.r7633

**Published:** 2015-02-11

**Authors:** Bruno Botta

**Affiliations:** 1 Department of Chemistry and Pharmaceutical Technology, Sapienza University of Rome, Rome, Italy

To identify drug candidates against Ebola virus infections is surely an urgent need, especially in light of recent virus outbreaks registered mostly in Africa. In this respect, Velijkovic's  article is presented in a timely manner and offers a fast and reliable opportunity to screen among large databases to reposition old drugs against Ebola.

The experimental design relies on a consolidated methodology, developed by some of the authors and successfully applied in multiple projects. Overall, the manuscript is clear and few minor editing would be necessary, in my personal opinion, to improve its consistency.

In Materials and Methods, a dataset of 152 drugs that counteract Ebola virus *in vitro* and *in vivo* has been selected as training set. However, it seems that an inconsistency does exist within this number. As authors have reported, these anti-Ebola drugs have been identified by Madrid (24 molecules) and Kouznetsova (53 molecules and 95 weaker drugs). Accordingly, the total number of FDA-approved drugs identified in these studies is 172. Why authors used a smaller set of 152? Is there any structural redundancy? A clarification of this discrepancy would improve the reproducibility of the work.

Finally, if I understood properly authors have selected more than 500 molecules (including FDA-approved and experimental drugs) as anti-Ebola candidates by means of *in silico *screening and suggest that further *in vitro/in vivo* tests should be performed on these molecules. In my opinion, this number is still too large for enabling efficient and fast *in vitro *and/or *in vivo* assays. Experimental testing of this set would require significant efforts. Just for comparison, the number of candidates selected in silico by authors is about half of those selected by Madrid by means of HTS (ref 2). Is there a way to prioritize small molecules by using the EIIP/AQVN-based approach, and to provide a lower number of compounds to be submitted to experimental evaluation? Authors should comment on this point, because the advantage of using the EIIP/AQVN-based screening in silico appears to be limited in the current version of the manuscript.

I have read this submission. I believe that I have an appropriate level of expertise to confirm that it is of an acceptable scientific standard.

# Peer review: Referee response for version 1

**DOI:** 10.5256/f1000research.6544.r7552

**Published:** 2015-02-11

**Authors:** Patrick Butaye

**Affiliations:** 1 School of Veterinary Medicine, Ross University, Basseterre, Saint Kitts and Nevis

This manuscript deals with the *in silico* analysis of molecules for their activity against Ebola Virus (EBV). They started from a reference library of compounds who have previously demonstrated *in vitro* and/or *in vivo *activity against EBV and analyzed these compounds by the determination of their EIIP and AQVN. With these data, they scanned a larger collection of compounds with unknown activity against EBV and selected possible candidates to test for their activity *in vitro *and/or *in vivo*. This is a straight forward manuscript however it may be better structured. The part of the M&M dealing with the EIIP and AQVN is more appropriate to go into the introduction since this is background information of the calculation. For clarity of the manuscript is also better to separate the results section as it is difficult to follow now. Start with the analysis of the compounds with known activity, the two datasets, and then proceed with results from the unknown dataset. Then in the discussion, the different products of interest may be evaluated. This will largely increase the readability. Upon the antibiotics, it would be good to elaborate a bit on how they work on EBV, since common sense tells that antibiotics do not work on viruses. A better explanation on how these products may interact and inhibit/kill EBV would also be good.

I have read this submission. I believe that I have an appropriate level of expertise to confirm that it is of an acceptable scientific standard.
